# Self-management research of asthma and good drug use (SMARAGD study): a pilot trial

**DOI:** 10.1007/s11096-017-0495-6

**Published:** 2017-06-09

**Authors:** Esther Kuipers, Michel Wensing, Peter de Smet, Martina Teichert

**Affiliations:** 1 Department of IQ Healthcare, Radboud Institute for Health Sciences, Radboud University Medical Centre, PO Box 9101, 6500 HB Nijmegen, The Netherlands; 2 Apotheek Rosmalen, Berlicum & Empel, ’s-Hertogenbosch, The Netherlands; 3 Department of General Practice and Health Services Research, University Hospital Heidelberg, Heidelberg, Germany; 4 Department of Clinical Pharmacy, Radboud Institute for Health Sciences, Radboud University Medical Centre, Nijmegen, The Netherlands; 5 Department of Clinical Pharmacy & Toxicology, Leiden University Medical Center, Leiden, The Netherlands

**Keywords:** Adherence, Asthma, Inhalation corticosteroid maintenance therapy, Netherlands, Pharmacotherapy, Pharmacy practice research

## Abstract

*Background* Community pharmacists play an important role in supporting patients for optimal drug use. *Objective* To assess the effectiveness of monitoring in asthma patients with inhaled corticosteroids (ICS) on disease control. *Setting* Asthma patients using ICS were invited from two intervention (IG) and two control pharmacies (CG). *Method* Participating patients completed questionnaires at the study start and at 6-month follow-up, including the Control of Allergic Rhinitis and Asthma Test (CARAT) questionnaire. IG patients completed the CARAT questionnaire every 2 weeks and received counselling on disease management, ICS adherence, and inhalation technique when scores were suboptimal, deteriorating, or absent. For Turbuhaler users, additional electronic monitoring (EMI) was available, with daily alerts for ICS intake. *Main outcome measure* As the primary outcome, CARAT scores at follow-up were compared between IG and CG using linear regression. As secondary outcome, refill adherence was compared using logistic regression. *Results* From March to July 2015, we enrolled 39 IG and 41 CG patients. At follow-up, CARAT scores did not differ between IG and CG (−0.19; 95% confidence interval [CI], −2.57 to 2.20), neither did patient numbers with ICS adherence >80% (0.82; 95% CI, 0.28–2.37). Among EMI users, CARAT scores did not differ, but ICS adherence >80% showed a 4.52-fold increase (95% CI, 1.56–13.1) compared with EMI nonusers. *Conclusion* Among community-dwelling asthma patients, pharmacist monitoring did not affect CARAT scores, but EMI use showed improved ICS refill adherence.

## Impacts on practice

- Dutch community pharmacists play a role in monitoring asthma patients for effective use of maintenance medication.
- The use of the CARAT questionnaire to report disease control every 2 weeks is feasible in asthma patients.
- Electronic monitoring improves ICS adherence in astma patients.
- Disease stability was not influenced by tailored pharmacist interventions on CARAT scores every two weeks compared to usual care.

## Introduction

An estimated 235 million people worldwide suffer from asthma [1]. Maintenance therapy with inhaled corticosteroids (ICS) has played a central role in gaining and maintaining asthma control [2]. Interventions by community pharmacists reportedly improve inappropriate inhalation techniques, asthma control, patient-reported asthma-related functional status, asthma severity, and symptoms [3].

At present, pharmacists usually intervene during dispensing visits [4, 5]. However, some patients may develop imperfect asthma control, and poorly adherent patients may not show up for subsequent dispensing. Timely interventions targeted at patients with suboptimal disease control may be effective in preventing exacerbations and deteriorating disease control between dispensing visits [6–8]. To promote such interventions, tools are needed to continuously monitor the process of drug intake and disease control. Ideally, patients and pharmacists should cooperate in monitoring symptoms and actively manage disease control.

The available tools for prospective monitoring include questionnaires on asthma control and electronic devices measuring drug intake [9–12]. One example of the former is the Control of Allergic Rhinitis and Asthma Test (CARAT) questionnaire, which has been validated for disease control of asthma and allergic rhinitis [13–17]. The use of medication can also be measured based on electronic monitoring of the intake of inhalation medication (EMI); that has been suggested as a well-validated means of measuring patterns of medication use [10, 11, 18]. Electronic monitoring has been widely studied for many years [19–23], and it was recently shown to have a positive impact on the use of inhalation medication [10, 24].

Regular employment of the CARAT questionnaire for patient-reported monitoring and continuous utilization of EMI enable monitoring of patients’ disease control and medication use. However, the usefulness of that information toward providing timely, tailored interventions in clinical practice is largely unknown. In theory, health-care providers can apply an individualized, data-driven approach for tailored interventions. For example, some patients could be helped by simplification of the dosing regimen or by practical advice linking medication intake to robust daily habits. Conversely, patients with intentional non-adherence could benefit from motivation and information about the disease, drug effects, and side effects; patients with a poor inhalation technique may benefit from improved inhaler use [7].

## Aim of the study

In this pilot study, we investigated the effects of tailored pharmacists’ interventions on patients’ asthma control by prospective monitoring with patient-reported CARAT scores compared with a control group receiving usual care. Secondary objectives were the effectiveness of the intervention on ICS adherence and on the number of exacerbations. All outcomes were additionally analysed with respect to the use of EMI in a planned subgroup analysis.

## Ethics approval

The study protocol was approved by the Ethical Committee of the Radboudumc Nijmegen (approval number, 2015-1569), and the trial was registered at The Netherlands National Trial Register (identifier, NTR5063). All procedures performed in studies involving human participants were in accordance with the ethical standards of the institutional and/or national research committee and with the 1964 Helsinki declaration and its later amendments or comparable ethical standards. Informed consent was obtained from all individual participants included in this study.

## Method

### Design and setting

This clustered controlled clinical trial was conducted between March 2015 and January 2016 in four community pharmacies in a rural area of the southern Netherlands. Dutch pharmacists have a professional and legal responsibility for the drug treatment of their patients [25]. As most patients in the Netherlands visit one community pharmacy, pharmacists usually possess the complete medication histories of their patients [26–28].

The four community pharmacies had comparable care structures: they all worked according to a certified quality management system and cooperated well with general practitioners (GPs) in structured pharmacotherapy circles (on average six GPs per pharmacy). Concealed from the patients, two pharmacies were designated as an intervention group (IG) with the intervention programme (see below). We made this choice to achieve equal practice procedures in each group. Patients in the two other pharmacies received usual pharmaceutical care–control group (CG).

### Patient inclusion

During regular pharmacy visits or by telephone, patients were invited to participate in this study when meeting the following selection criteria according to their pharmacy database: (1) age 18–60 years; and (2) current user of asthma maintenance medication. The medication included ICS or a combination of ICS and long-acting beta-agonist (LABA); the Anatomic Therapeutic Chemical (ATC) codes were R03BA, R03AK06, and R03AK07 [29], with at least two prescriptions of ICS in the previous 6 months. A current diagnosis of asthma and no (con)current chronic obstructive pulmonary disease was verified by information from the patient and the GP. Patients were included if they spoke, read, and wrote Dutch. Informed consent was obtained from all individual participants included. The follow-up lasted for 6 months after patient inclusion.

EMI could be used for inhalation medication with budesonide and formoterol (Turbuhaler) [24]. The device was connected by Bluetooth^®^ to an application on the patient’s smartphone and registered every inhalation. The application was provided at no expense for the patient, and patients voluntarily shared their data with the pharmacist. Data were registered in a safe manner and provided only to the patient and pharmacist. Information on medication use became visible in the application (for up to 7 days) and a personal web portal (up to 30 days) for both the patient and pharmacist. The application reminded patients twice daily to take their medication. Both IG and CG patients were eligible for this programme if they met additional inclusion criteria: (1) at least two prescriptions of budesonide or formoterol Turbuhaler (ATC code R03AK07) (26) in the previous 6 months; (2) access to a smartphone; and (3) possessed skills to use the Internet.

### Interventions

#### Training of health-care professionals

IG pharmacists and pharmacy assistants received additional training on asthma symptoms, treatment, possible side effects, and smoking cessation. Furthermore, they underwent 3-h training in interviewing techniques, with a focus on exploring a patient’s ambivalence or readiness for behavioural change. They were also trained to give inhalation instructions and to use the CARAT questionnaire for monitoring asthma control. Pharmacists and assistants from all pharmacies received information about the EMI; however, only IG pharmacists used the monitoring information of their patients.

#### Intake and counselling session

CG patients received standard care and checks on their inhalation technique; instructions were provided only at their own request. IG patients received an intake session as a one-to-one private counselling session with a trained pharmacist or pharmacy assistant. Depending on their needs and health literacy during those sessions, patients received tailored education on the following: asthma pathophysiology (symptoms and triggers); self-management (e.g. lifestyle advice); smoking cessation (if the patient was a current smoker); and the effects of their asthma medication. For this purpose, information from official pharmacist guidelines on asthma and patient counselling during dispensing were used [30, 31]. Different elements of inhalation medication use were discussed, such as dosing and time of intake, the importance of adherence to maintenance therapy, and problems with adherence or experienced side effects and their prevention (e.g. rinsing the mouth after inhalation, good inhalation technique). In addition, the inhalation technique was checked with the patient using a demonstration inhaler unit.

#### Timely, tailored interventions based on CARAT scores

During follow-up, the CARAT questionnaire was freely available for IG patients as a smartphone and tablet application. IG patients were instructed to download the application and received a reminder to complete and send the score every 2 weeks to the pharmacist. Via their personal e-mail, patients received graphic results of the CARAT scores they had provided; the results were presented as the scores for both domains (lower and upper airways) and the total score, and were sent by e-mail every 2 weeks. This information offered additionally self-monitoring options and insights for the IG patients.

If a CARAT score was not received within 16 days, the score signalled disease instability (total CARAT score ≤10) [15, 16], or the CARAT score deteriorated substantially (≥4 points) [15, 16], the IG pharmacist contacted IG patients by e-mail or phone to identify the reasons. According to the patient’s individual situation, the pharmacist offered a tailored intervention. For IG patients in the EMI group, the pharmacist used the EMI data to check actual drug use.

### Measures and outcomes

#### Measurement of disease control by CARAT questionnaire

The primary outcome of the study was asthma control, measured by the CARAT questionnaire, compared between IG and CG patients. The CARAT is a 10-item questionnaire developed to measure disease control of asthma and allergic rhinitis [13–16]. The first nine questions offer scores of 0 (complete absence of control) to 3 points. The last question on increased medication use the previous week has three response options (‘never’ = 3 points, ‘less than 7 days’ = 2 points, ‘more than 7 days’ = 0 points) and an option ‘I do not take any additional medication to control my asthma,’ which was also attributed 3 points. The CARAT score was calculated as the sum of the scores for all questions and ranged from 0 to 30 [14].

#### Secondary outcomes

Secondary outcomes addressed the number of exacerbations and differences in medication adherence to ICS, measured by the Medication Adherence Report Scale (MARS-5) and by ICS refill data. Exacerbations were counted using pharmacy dispensing data of the Dutch Foundation for Pharmaceutical Statistics (SFK) [32] as well as 6 months prior to the study start and 6 months during the study period. In accordance with prevailing clinical practice guidelines, we defined an exacerbation as treatment with a course of a systemic corticosteroid (ATC codes H02AB06 and H02BA07) [29] at a dose of at least 20 mg or higher for 5–14 days [33].

IG and CG patients completed the MARS-5-questionnaire at the beginning and end of the study. The MARS-5 questionnaire is a five-item self-report measure of medication adherence for rating the frequency of different types of non-adherent behaviour [34, 35]. We calculated medication adherence from ICS refill data as the proportion of days covered (PDC) by maintenance therapy with ICS [36]—whether or not in fixed combination with an LABA (ATC codes R03BA, R03AK06, R03AK07) [29]—from routinely collected dispensing data of the SFK. We calculated PDC percentages for 6 months prior to the study start and at study end for 6 months during the study period.

In a planned subgroup analysis, we additionally compared all measures between patients with and without EMI.

### Sample size

We calculated the minimal sample size for the ability to simultaneously detect a difference of 4 points [16] in CARAT scores at an assumed standard deviation (SD) of 7 and difference in medication adherence of 15% in medication possession rate (SD = 20%) between the study end and start, with 80% power at the 5% two-sided significance level. Allowing for a dropout rate of 5%, we aimed at enrolling 80 patients [13, 14].

### Statistical analysis

Using linear regression analysis, we compared the CARAT scores and mean medication adherence at follow-up between the IG and CG patients, adjusted for the subject’s measurement at the study start in addition to age and sex. As neither the PDC nor the MARS-5 scores and the number of oral corticosteroid courses fulfilled the requirements for linear regression analysis (e.g. normal distribution), we used logistic regression analysis for dichotomized cut-off models, adjusted for the subject’s age, sex, and status at the study start. We performed all analyses using IBM Corp SPSS statistics, Chicago IL, USA, version 23.

## Results

**Fig. 1:**
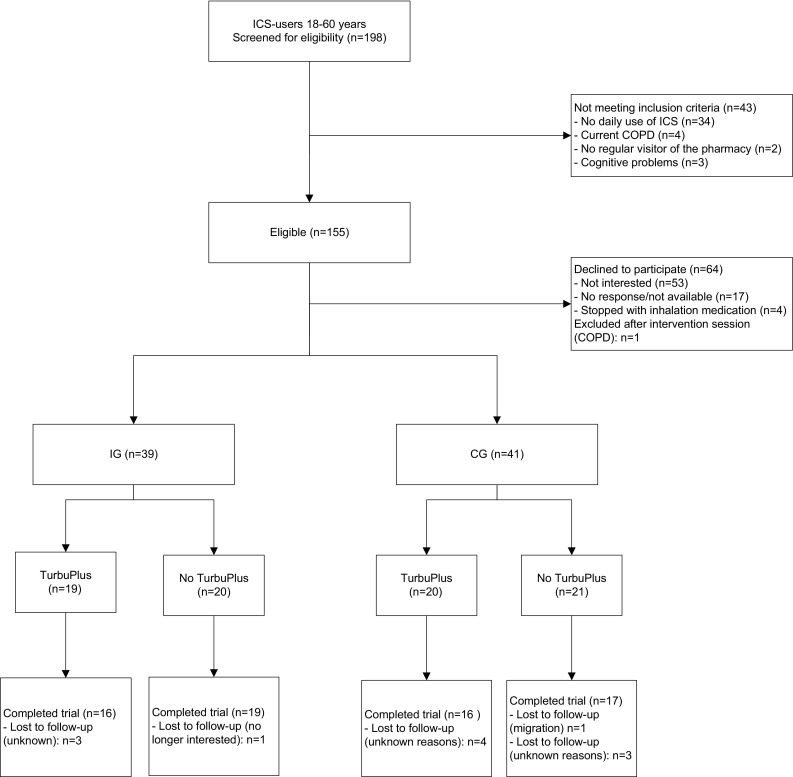
Flowchart participants during the study

In the four pharmacies, 198 patients were screened for eligibility, of whom 155 (78.3%) met all the inclusion criteria (Fig. 1). In all, 80 patients (52%) agreed to participate: 41 in the CG and 39 in the IG. The two study groups were comparable regarding baseline characteristics, including type of inhaled corticosteroids (Table 1); however, the mean age of IG patients was higher than that of CG patients: 44.95 versus 39.34 years; *P* = 0.015. The trial was completed by 68 patients; 12 patients were lost to follow-up, largely for unknown reasons.

**Table 1:** Baseline characteristics

Parameter	Intervention group (n = 39)	Control group (n = 41)
Female sex [n (%)]	23 (59.0)	27 (65.9)
Age [years; mean (SD)]	44.95 (8.43)	39.34 (11.48)
Asthma, duration [years; mean (SD)]	23.9 (17.2)	20.9 (14.3)
Number of exacerbations treated by oral corticosteroid courses 6 months before inclusion [mean (range)]	0.13 (0; 4)	0.02 (0; 1)
Smoking status:		
Current [n (%)]	9 (22.5)	4 (9.8)
Earlier [n (%)]	8 (20.0)	12 (29.3)
Never [n (%)]	22 (55.0)	24 (58.5)
Electronic monitoring [n (%)]	19 (48.7)	20 (48.8)
CARAT total score [points (95% CI)]	20.36 (17.96–22.76)	21.29 (19.43–23.15)
CARAT upper airways score [points (95% CI)]	7.46 (6.22–8.70)	8.27 (7.26–9.27)
CARAT lower airways scores [points (95% CI)]	12.90 (11.24–14.56)	13.02 (11.74–14.31)
MARS-5 score [points (95% CI)]	20.79 (19.76–21.83)	21.22 (20.05–22.39)
Adherence ICS with dispensing data [% PDC (95% CI)]	72.58 (65.46–79.70)	84.73 (77.57–91.88)

Among the 39 IG patients, 27 completed all 13 measurements during follow-up. Owing to deteriorating CARAT scores, 44 interventions were performed in 24 (61.5%) of the IG patients, with a maximum of four interventions for one patient (Table 2).

**Table 2:** Pharmacist interventions

Situation	Pharmacist intervention	Frequency
Decreased score on CARAT-domain upper airways	Inquire about actual hay fever complaints and recommended the use of an oral, ocular or nasal antihistamines or nasal corticosteroids	32 times
Low adherence scores	Tailored advice to eventual barriers to chronic drug use or fear of ICS side effects or to patients’ poor knowledge of asthma disease. Discuss the importance of medication adherence	4 times
CARAT-score decreased substantially, possible overuse of short acting beta agonists (SABA, use of ≥ 3 times a week)	Contact with patient to explore actual symptoms and possible reasons. Invitation for visiting the pharmacy for a check of the inhalation technique. Contact with prescriber to discuss switch of medication (e.g. another nasal corticosteroid)	4 times
Persisting symptoms, despite interventions and adherent use of ICS	Referral to the general practitioner for evaluation of persisting symptoms	2 times
CARAT-score ≤10; indicating a possible exacerbation	Referral to the general practitioner for examination of a possible exacerbation and prescription of rescue medication, if needed	2 times

At baseline, the mean CARAT scores were comparable between the IG (20.36 points) and CG (21.29 points). In multivariate regression analysis, the total CARAT scores at follow-up did not differ between the IG and CG (Table 3): mean estimated difference, –0.19 for the total score; 95% confidence interval (CI), −2.57 to 2.20). Likewise, the CARAT scores for the upper airways (–0.22; 95% CI, −1.01 to 1.44) and lower airways (−0.62; 95% CI, −2.30 to 1.06) did not vary. We observed no difference between the groups for the outcomes for medication adherence: the probability of having a period covered by drug use >80% did not vary between IG and CG (Odds Ratio, OR 0.82; 95% CI, 0.28–2.37).

**Table 3:** Differences in outcome measures between intervention and control group at follow up

Outcome measure	Difference
CARAT total score (95% CI)^a^	−0.19 (−2.57 to 2.20)^a^
CARAT upper airways score (95% CI)^a^	0.22 (−1.01 to 1.44)
CARAT lower airways scores (95% CI)^a^	−0.62 (−2.30 to 1.06)
Period covered by drug dispensings >80% (95% CI)^b^	0.82 (0.28–2.37)
MARS-5 score > 20 (95% CI)^b^	0.55 (0.15–2.05)
At least one oral corticosteroid short course^b^	No corticosteroid short courses in control group

^a^ Linear regression analysis, adjusted for age, sex and baseline score, CI = Confidence Interval)

^b^ Logistic regression analysis, adjusted for age, sex and baseline score

The probability of achieving a score >20 on the MARS-5 questionnaire (28) at the study end did not differ between the two groups (0.55; 95% CI, 0.15–2.05). Finally, no differences between IG and CG were found for the number of exacerbations, measured by oral corticoid courses.

A planned subgroup analysis was performed for the 39 patients with EMI compared with the 41 without EMI. Those groups did not differ in terms of baseline characteristics, except for a higher mean age of EMI patients: 44.08 years versus 40.17 years; *P* = 0.001 (Table 4). In the EMI subgroup, refill adherence >80% showed a 4.52-fold increase: 95% CI, 1.56–13.1 compared with no EMI use. We observed no differences among the other measures (Table 5).

**Table 4:** Baseline characteristics for subgroups with and without EMI

Parameter	EMI-group (n = 39)	No EMI- group (n = 41)
Female sex [n (%)]	21 (53.8)	29 (70.1)
Age [years; mean (SD)]	44.08 (6.93)	40.17 (12.71)
Asthma, duration [years; mean (SD)]	23.50 (15.49)	21.32 (16.20)
Number of exacerbations treated by oral corticosteroid courses 6 months before inclusion [mean (range)]	0.10 (0–2)	0.12 (0–1)
CARAT total score [points (95% CI)]	20.95 (18.62–23.27)	20.73 (18.78–22.68)
CARAT upper airways score [points (95% CI)]	8.00 (6.81–9.19)	7.76 (6.68–8.83)
CARAT lower airways scores [points (95% CI)]	12.95 (11.38–14.51)	12.98 (11.59–14.36)
MARS-5 score [points (95% CI)]	21.08 (19.97–22.18)	20.95 (19.84–22.06)
Adherence ICS with dispensing data [% PDC (95% CI)]	82.38 (75.47–89.28)	75.42 (67.74–83.08)

**Table 5:** Differences in outcome measures compared between patients with and without electronic monitoring device at follow up

Outcome measure	Difference
CARAT total score (95% CI)^a^	1.49 (−0.82 to 3.80)
CARAT upper airways score (95% CI)^a^	0.95 (−0.20 to 2.10)
CARAT lower airways scores (95% CI)^a^	0.52 (−1.12 to 2.17)
Period covered by drug dispensing > 80% (95% CI)^b^	**4.52 (1.56–13.1)**
MARS-5 score >20 (95% CI)^b^	2.13 (0.60–7.55)
At least one oral corticosteroid short course^b^	3.40 (0.25–46.50

Statistically significant outcomes are printed in bold

^a^ Linear regression analysis, adjusted for age, sex and baseline score

^b^ Logistic regression analysis, adjusted for age, sex and baseline score

## Discussion

In this study, we found that additional timely, tailored pharmacist interventions did not increase asthma control or ICS adherence compared with usual care. With EMI, we recorded effects on refill adherence but not on the CARAT or MARS-5 scores.

Though at first sight these results appear disappointing, a number of mitigating considerations exist. First, this investigation was established as a pilot study to determine the usefulness and feasibility of patient-reported monitoring in measuring asthma control over time. Some studies have investigated community pharmacist interventions to improve asthma control; however, disease control was mainly assessed using the Asthma Control Questionnaire or Asthma Control Test, not the CARAT questionnaire [2, 3]. The number of eligible patients willing to participate in the present study was just sufficient to detect a difference in CARAT scores of 4 points between the study groups; that is considered a clinically relevant score, according to the CARAT developers [16]. At baseline, little was known about the CARAT scores of community-dwelling asthma patients in primary care. Our study showed high CARAT scores—an average of 21 points—for this population at study start. Hitherto, CARAT scores have been measured monthly, and little has been known about their development over time. The measurement of CARAT scores every 2 weeks was feasible in the IG and enabled regular pharmacist-patient contacts between dispensing visits.

In the planned subgroup analysis for EMI, ICS refill adherence >80% was 4.52-fold (95% CI, 1.56–13.1) that of EMI non-users. When including only subjects with CARAT scores below 23 points at the study start, the OR of achieving higher CARAT scores at the study end was 2.87 (95% CI 0.61–13.6) for the EMI group compared with the non-EMI group. This finding suggests that poor asthma control due to underuse of maintenance therapy with ICS may be improved more effectively in this population by EMI than with a tailored pharmacist intervention. Regarding the difficulty in demonstrating the effects of tailored interventions on disease outcomes, the findings of the present study are not unique: a recent investigation about tailored counselling on health-related lifestyles in cardiovascular diseases also reported no effect on the primary outcome [37]. This suggests that for asthma patients in primary care, EMI may be sufficient for improving medication adherence; however, the effects on disease outcomes remain to be shown. Furthermore, selection bias cannot be fully excluded in the present study as patients voluntarily participated in the study and for EMI use if suitable. In general however, in the Netherlands all inhabitants are obliged to have a health care insurance, which gives access to all asthma medications. Therefore we do not expect selection bias from this cause for our findings.

The absence of spirometric confirmation of the asthma diagnosis could be considered a limitation. However, pharmacists do not generally have access to such data. Corresponding with clinical practice, an asthma diagnosis was initially assumed from the use of asthma medication; it was verified with the patient and information from the registration of contraindications in the computer system of the GP, if available. We did not dispose of information on comorbidities. Although asthma patients included were relatively young and patients’ age was comparable between the groups, we cannot fully exclude that we might have missed differences between the groups due to comorbidity. With regard to exacerbations, the use of short-term corticosteroid courses was low in both groups. A sub-analysis with pooled measures of both types of short-term courses did not achieve statistical significance. Finally, the use of EMI within both IG and CG groups may have influenced our intervention. However, in further analysis, we did not observe any interaction between the intervention and EMI use (*P* = 0.11 for a multiplicative interaction term).

## Conclusion

Our results did not show an effect of tailored pharmacist interventions on patient-reported disease control in a general asthma population compared with usual care. To support non-intentional non-adherence in this population, EMI may be effective; however, that strategy needs to be confirmed with greater patient numbers for a longer follow-up period for clinical outcomes.
